# Altered Taste Function in Young Individuals With Type 1 Diabetes

**DOI:** 10.3389/fnut.2021.797920

**Published:** 2022-01-12

**Authors:** Eulalia Catamo, Antonietta Robino, Davide Tinti, Klemen Dovc, Roberto Franceschi, Manuela Giangreco, Paolo Gasparini, Egidio Barbi, Vittoria Cauvin, Ivana Rabbone, Tadej Battelino, Gianluca Tornese

**Affiliations:** ^1^Institute for Maternal and Child Health, Scientific Institute for Research, Hospitalization and Healthcare Burlo Garofolo (IRCCS), Trieste, Italy; ^2^Center for Pediatric Diabetology, A.O.U. Città della Salute e della Scienza, Torino, Italy; ^3^Faculty of Medicine, University of Ljubljana, Ljubljana, Slovenia; ^4^Department of Endocrinology, Diabetes and Metabolism, University Children's Hospital, University Medical Centre Ljubljana, Ljubljana, Slovenia; ^5^Division of Pediatrics, S. Chiara General Hospital, Trento, Italy; ^6^Department of Medical Sciences, University of Trieste, Trieste, Italy; ^7^Division of Pediatrics, Department of Health Sciences, Università del Piemonte Orientale, Novara, Italy

**Keywords:** taste function, type 1 diabetes, pediatric samples, age at onset, glycemic control

## Abstract

Past studies on altered taste function in individuals with type 1 diabetes have yielded inconsistent results. We therefore evaluated taste recognition and possible association with personal and diseases characteristics in young individuals with type 1 diabetes and healthy controls. Taste recognition and intensity for 6-n-propylthiouracil (PROP), quinine, citric acid, sucrose, and sodium chloride were assessed using a filter paper method in 276 participants with type 1 diabetes and 147 healthy controls. Personal and clinical data were recorded for all participants during a baseline visit. Regression analysis was adjusted for sex, age, and standardized BMI. Overall, 47% of participants with type 1 diabetes vs. 63.5% of healthy controls recognized all tastes (*p* = 0.006). Moreover, a lower capacity for recognizing the bitter taste of PROP and the sour taste of citric acid was found in participants with type 1 diabetes compared to healthy controls (*p* = 0.014 and *p* = 0.003, respectively). While no significant effect of glycemic control on taste recognition was found, an association with lower age at onset emerged. Our findings suggest an impaired taste perception in individuals with type 1 diabetes, possibly linked to age at onset.

## Introduction

Taste perception is one of the most important factors influencing individual food preferences and eating habits with possible implications on health status (1–3). Recently, the study of taste function and its relationship with diseases, such as obesity or diabetes, has received increasing attention. Several reports have already described taste impairment in type 2 diabetes, suggesting a possible impact of this disorder on the ability to follow a controlled diet and thus reaching good glycemic control. Taste impairment in type 2 diabetes has also been related to micro- and macro-vascular complications of the disease (4–7).

Altered taste function is also reported in individuals affected by type 1 diabetes. Many years ago, hypogeusia involving all the four primary tastes (bitter, salty, sour, sweet) was described in adults with type 1 diabetes, significantly associated with type 1 diabetes duration and its complications such as peripheral neuropathy, proposing that the impairment could be a complication of the disease (8).

Changes in electrogustometric taste thresholds and in the gustatory anatomical structures were also reported (9). More recently, a significantly increased threshold for bitter, salty, sour, and sweet tastes was observed in 70 participants with type 1 diabetes compared to controls (10). Another recent study in 31 pediatric participants with uncomplicated type 1 diabetes shows a significantly lower ability to correctly identify bitter and sour tastes compared to healthy controls (11).

However, other studies showed no significant differences in taste function between participants with uncomplicated type 1 diabetes and healthy subjects (12, 13). Furthermore, there are conflicting reports on the associations between taste impairment and metabolic control, disease duration, and the presence of diabetes-related complications (8, 12).

Therefore, in the present study, we evaluated taste perception in young individuals with type 1 diabetes and healthy controls. In participants with type 1 diabetes, we also investigated the possible influence of personal and disease characteristics (e.g., puberty, age at onset, onset with ketoacidosis, disease duration, etc.) on taste function.

## Materials and Methods

### Subjects

We included 276 individuals with type 1 diabetes and 147 healthy controls.

Participants were recruited at Diabetes Units of IRCCS Burlo Garofolo (Trieste, Italy), Regina Margherita Children's Hospital (Torino, Italy), Santa Chiara Hospital (Trento, Italy), and UMC Ljubljana University Children's Hospital (Ljubljana, Slovenia) between October 2018 and December 2019.

Inclusion criteria were diagnosis of type 1 diabetes (14), age between 6 and 21 years, type 1 diabetes duration of at least 1 year. Participants with other types of diabetes (i.e., type 2 diabetes, monogenic diabetes, cystic fibrosis-related diabetes) were excluded.

Sex- and age-matched healthy controls were recruited from emergency department. They were not included if they had: type 1 or type 2 diabetes, obesity or other metabolic disorders, glycated hemoglobin (HbA1c) > 6% (>42 mmol/mol), family history of diabetes/obesity, as well as other diseases (e.g., respiratory infection) affecting smell or taste function. The ethics committee approved the protocol [CEUR-2018-Em-323-Burlo (Italy) and KME-0120-65/2019/4]. All participants and their parents (for participants aged <18 years) gave written informed consent/assent prior to enrolment.

### Personal and Clinical Data

In participants with type 1 diabetes, all data were collected during a follow-up visit (15). Personal information such as age, sex, and pubertal status was obtained. Puberty is defined as the presence of breast budding in girls and testicular volume of 4 ml in boys (16). The following clinical measurements collected on the day of the taste analysis were available: blood glucose concentration, HbA1c, insulin daily requirement, disease duration, height, weight, and BMI. Moreover, the following data from the type 1 diabetes onset were collected: age, HbA1c, blood glucose concentration at admission, presence of ketoacidosis, and insulin daily requirement at discharge.

In healthy controls, weight, height, and BMI were collected, and HbA1c was measured using DCA 2000 Analyzer System (Siemens, Munich, Germany).

In all participants, the standard deviation scores (SDS) of weight, height, and BMI were calculated according to WHO reference charts (17) using a software (Growth Calculator 3: http://www.weboriented.it/ghc3/).

### Taste Evaluation

Using a filter paper method, we evaluated the capacity to recognize the following compounds: PROP (0.0085 g/ml), quinine (0.0024 g/ml), citric acid (0.165 g/ml), sucrose (0.2 g/ml) and sodium chloride (0.058 g/ml) (18). Specifically, after receiving instruction by an expert administrator, each participant was asked to rinse the mouth with bottled filtered water, place the paper on the tongue and recognize the correct taste among sweet, bitter, salt, sour (4-alternative forced choice). A possible choice was “I do not perceive any taste,” and this answer was considered a missing value. A binary variable was used to define taste recognition for each compound: “yes” if the subject correctly identifies the compound and “no” if the subject does not correctly identify the compound.

### Statistical Analysis

Descriptive statistics represent percentages, means, and standard deviations.

To evaluate taste recognition in type 1 diabetes participants and healthy controls, logistic regression analysis was performed. Gender, age, and standardized BMI were included as covariates in all models.

Linear or logistic regression models with age, gender, standardized BMI, and disease duration were also performed in participants with type 1 diabetes to test the association between personal or clinical characteristics and taste recognition.

Glycemic control was evaluated grouping participants with type 1 diabetes based on HbA_1c_ levels as follow: optimal control (OC) [HbA1c <7.5% (<58 mmol/mol), given previously HbA1c target from ISPAD 2014], intermediate control (IC) [HbA1c 7.5–8.5% (58–70 mmol/mol)], and poor control (PC) [HbA1c > 8.5% (>70 mmol/mol)] (19).

Statistical significance was set at a *p*-value ≤ 0.05. All statistical analyses were performed with R software (www.r-project.org).

## Results

### Sample Characteristics

Sample characteristics of participants are reported in Table 1. No age and gender differences were found comparing healthy controls and type 1 diabetes participants. The age range was from 6 to 20 years with a mean and standard deviation of 12.3 ± 3.4 in controls and 12.8 ± 3.3 in type 1 diabetes subjects. A significant difference emerged among healthy subjects and participants with type 1 diabetes for standardized BMI (−0.36 ± 1.1 vs. 0.08 ± 1.1, *p* = 0.0001).

**Table 1:** Sample characteristics of type 1 diabetes subjects and healthy subjects.

	**Healthy subjects (*n* = 147)**	**Type 1 diabetes subjects (*n* = 276)**	***p*-value**
Gender (% females)	55%	47%	0.14
**Data at taste evaluation**			
Age, years (mean ± sd)	12.3 ± 3.4	12.8 ± 3.3	0.15
Standardized BMI, SDS (mean ± sd)	−0.36 ± 1.1	0.08 ± 1.1	0.0001
Puberty (% yes)	61%	76%	0.13
HbA1c, % (mean ± sd)	5.5 ± 0.2	7.8 ± 1.1	<2 × 10^−16^
HbA1c, mmol/mol (mean ± sd)	37 ± 2	62 ± 12	
Glycemia, mg/dL (mean ± sd)	–	180 ± 88	–
Insulin daily requirement, U/kg/day (mean ± sd)	–	0.75 ± 0.25	–
Disease duration (years) (mean ± sd)	–	5.4 ± 3.6	–
**Data at diabetes onset**			
Age, years (mean ± sd)	–	7.5 ± 3.8	–
HbA1c, % (mean ± sd)	–	11.2 ± 2.3	–
HbA1c, mmol/mol (mean ± sd)		99 ± 24	
Glycemia at admission, mg/dL (mean ± sd)	–	457 ± 167	–
Ketoacidosis (% yes)	–	43%	–
Insulin daily requirement at discharge, U/kg/day (mean ± sd)	–	0.59 ± 0.29	–

*As possible, significant differences among type 1 diabetes subjects and healthy subjects were assessed by T-test for quantitative variables and Chi-squared test for categorical variables*.

Mean HbA1c in participants with type 1 diabetes was 7.8 ± 1.1% - 62 ± 12 mmol/mol (range 5.5–13.4% - 37–123 mmol/mol); all healthy controls have A1c below 6%. 39.5% of type 1 diabetes individuals achieved OC, 37.5% IC and 23% PC.

Of the 276 type 1 diabetes participants, 76% were in puberty, and 43% presented ketoacidosis at the onset.

### Taste Evaluation Among Participants With Type 1 Diabetes and Healthy Controls

Overall, 47% of participants with type 1 diabetes vs. 63.5% of healthy controls recognized all tastes (*p* = 0.0006) (Table 2). Moreover, PROP and citric acid recognition was less common in participants with type 1 diabetes (*p* = 0.014 and *p* = 0.003, respectively). No significant differences emerged for quinine and sodium chloride recognition.

**Table 2:** Taste recognition in healthy subjects and type 1 diabetes subjects.

	**Healthy subjects (*n* = 147)**	**Type 1 diabetes subjects (*n* = 276)**	*p*-value* OR (CI)
**Taste recognition (% yes)**			
PROP	87%	77%	**0.014** 2.1 (1.2–3.9)
Citric acid	84%	72%	**0.003** 2.2 (1.3–3.7)
Quinine	75%	70%	0.244 1.4 (0.8–2.3)
NaCl	89%	85%	0.273 1.4 (0.7–2.7)
All	63.5%	47%	**0.0006** 2.1 (1.4–3.2)

“*All” refers to an overall taste recognition in which subjects that correctly identify all compounds were compared to others*.

^*^p-value from logistic regression analysis with sex, age, and standardized BMI as covariates.

*In bold are shown significant results*.

Since only six subjects (4 individuals with type 1 diabetes and two healthy controls) did not recognize sucrose, data on sucrose recognition were excluded.

### Association of Taste Recognition With Personal and Health Parameters in Type 1 Diabetes Participants

No significant differences in taste recognition were found among participants with type 1 diabetes divided by HbA1c values, and no significant effect of other tested variables (including puberty, ketoacidosis at onset, disease duration, median HbA1c over the last year) was detected.

Regression analysis showed that, irrespective of disease duration, earlier age at onset (defined as <6 years of age) was significantly associated with decreased overall taste recognition (*p* = 0.048), as well as PROP and quinine recognition (*p* = 0.027 and *p* = 0.015, respectively).

As reported in Table 3, the percentage of type 1 diabetes participants with type 1 diabetes recognizing tastes was lower among those with age at onset <6 years.

**Table 3:** Taste recognition accordingly age at onset.

	**Age at onset of type 1 diabetes subjects**		
	** <6 years (*n* = 100)**	**≥6 years (*n* = 174)**	*p*-value^*^ OR (CI)
**Taste recognition (% yes)**			
PROP	65%	83%	**0.025** 3.4 (1.2–10.1)
Citric acid	68%	73%	0.144 2.1 (0.8–5.5)
Quinine	62%	74%	**0.015** 3.8 (1.3–11.6)
NaCl	82%	87%	0.275 0.5 (0.1–1.7)
All	38%	52%	**0.048** 2.3 (1.1–5.5)

* *p-value from logistic regression analysis with sex, age, standardized BMI and disease duration as covariates*.

*In bold are shown significant results*.

## Discussion

In the present work, we compared taste recognition between young individuals with type 1 diabetes and healthy subjects. We identified significantly reduced overall taste perception, the bitterness of PROP, and the sour of citric acid in participants with type 1 diabetes. To date, findings on the relationship between type 1 diabetes and taste function are limited and controversial. Some studies report a difference in all four primary tastes (8, 10), others only in some taste qualities (11, 20), while others report no differences (12, 13). Our results substantiate findings that report an alterated PROP and citric acid perception in individuals with type 1 diabetes (10, 11). However, we did not find differences in quinine and NaCl recognition, unlike past studies conducted in both adult (8, 10, 20) and pediatric participants (11). Age and clinical characteristics of the enrolled participants could be among the reasons for the contradictory published results. Moreover, differences in the methods used to measure taste recognition across the studies may represent another possible confounder.

The mechanisms underlying taste dysfunction in diabetes are still unclear. Hyperglycemia has been indicated as one of the possible factors; it can induce a concentration-dependent impairment of taste perception (21), and long-standing hyperglycemia was associated with microvascular complications, such as neuropathy. In turn, peripheral nerve injury associated with neuropathy may involve lingual nerves, leading to gustatory impairments (8, 22). However, our data do not support this hypothesis in pediatric age with the mean disease duration of just over 5 years since HbA1c level at onset and at the time of the test was not associated with taste recognition.

Inflammation of oral mucosa may be another possible cause of the taste dysfunction observed in type 1 diabetes (23). As also described for dysgeusia associated with COVID-19 (24), inflammatory cytokines can trigger apoptosis and may cause abnormal turnover and loss in taste buds and ultimately the development of taste dysfunction.

Consistent with previous findings (12, 13), we did not find an association between taste perception and other type 1 diabetes characteristics (i.e., disease duration, puberty, ketoacidosis at onset, etc.). Otherwise, in the present work, we found an association between altered taste function and decreased age at onset, independently of diabetes duration. Although no guidelines consider the age of onset as a risk stratifier, it could be a proxy for several important factors related to type 1 diabetes, such as variations in autoimmune mechanisms. For example, early-onset type 1 diabetes is associated with the presence of other autoimmune diseases, higher insulin antibodies value, lower initial insulin reservoir, and higher insulin requirements 1 year after diagnosis (25). In an extensive study of 27,195 individuals with type 1 diabetes, age at onset also presented a critical determinant of survival and cardiovascular outcomes (risk of coronary heart disease and acute myocardial infarction) (26). Therefore, early onset may be more harmful than late-onset disease, with the worst prognosis and higher risk of related complications (26). Additionally, children developing type 1 diabetes in early childhood are more likely to score relatively poorly on cognitive tests, independent of diabetes duration. Children with onset before the age of 7 years are found to have mild central brain atrophy and significant differences in intellectual performance in adulthood (27). Taste recognition is also thought to be associated with cognitive function, although studies have been focused on the elderly (28, 29). Based on this evidence, we can speculate that differences in cognitive ability may also contribute to taste recognition observed in type 1 diabetes.

This work has some limitations. Our study protocol precludes the possibility of evaluating the potential link between taste alteration and type 1 diabetes-associated complications. Moreover, the lack of information on inflammation data or cognitive function does not allow to confirm cited studies on the mechanism responsible for taste alteration. Furthermore, while most of the literature has focused on sweet taste impairment in diabetes (4, 10), we did not evaluate this taste modality. Moreover, we did not assess the possible role of other factors that may influence taste function, as well as genetic polymorphisms in taste receptor genes (30), oral microbiota (31) and hormonal fluctuation throughout the menstrual cycle (32).

Despite these limitations, this study is, to our knowledge, the largest study so far documenting taste alteration in young individuals with type 1 diabetes.

In conclusion, taste impairment in individuals with type 1 diabetes was possibly related to age at onset. Further studies evaluating the actual mechanisms underlying taste changes in type 1 diabetes and its link with age at onset are warranted.

## Data Availability Statement

The raw data supporting the conclusions of this article will be made available by the authors, without undue reservation.

## Ethics Statement

The studies involving human participants were reviewed and approved by Comitato Etico Unico Regionale (CEUR-2018-Em-323-Burlo). Written informed consent to participate in this study was provided by the participants' legal guardian/next of kin.

## Author Contributions

EC, AR, and GT designed research, wrote the manuscript, and take responsibility for the contents of the article. EC, AR, and MG analyzed data or performed statistical analysis. EC, AR, DT, KD, and RF performed data collection. PG, EB, VC, IR, and TB contributed to the discussion and reviewed the manuscript. All authors read and approved the final manuscript.

## Funding

This work was supported by ISPAD-JDRF Fellowship 2017 and Institute for Maternal and Child Health IRCCS Burlo Garofolo, Trieste, Italy (RC 14/16) to GT, and by Italian Ministry of Health (GR-2019-12369573) to AR. KD and TB were supported in part by the Slovenian Research Agency Grant P3-0343.

## Conflict of Interest

The authors declare that the research was conducted in the absence of any commercial or financial relationships that could be construed as a potential conflict of interest.

## Publisher's Note

All claims expressed in this article are solely those of the authors and do not necessarily represent those of their affiliated organizations, or those of the publisher, the editors and the reviewers. Any product that may be evaluated in this article, or claim that may be made by its manufacturer, is not guaranteed or endorsed by the publisher.
